# Enhancing Diagnostic Accuracy of Ophthalmological Conditions With Complex Prompts in GPT-4: Comparative Analysis of Global and Low- and Middle-Income Country (LMIC)–Specific Pathologies

**DOI:** 10.2196/64986

**Published:** 2025-06-30

**Authors:** Shona Alex Tapiwa M'gadzah, Andrew O'Malley

**Affiliations:** 1 School of Medicine, University of St Andrews, North Haugh, St Andrews, KY16 9TF, United Kingdom, 44 01382384210

**Keywords:** artificial intelligence, AI, ophthalmology, clinical diagnostics, medical technology, data project, complex prompt, diagnostic accuracy, ophthalmological conditions, ophthalmological disorder, eyes, blindness, low- and middle-income countries, LMIC, low-income or middle-income economies, health care, LLMs, NLP, machine learning, statistical analysis, GPT-4

## Abstract

**Background:**

The global incidence of blindness has continued to increase, despite the enactment of a Global Eye Health Action Plan by the World Health Assembly. This can be attributed, in part, to an aging population, but also to the limited diagnostic resources within low- and middle-income countries (LMICs). The advent of generative artificial intelligence (AI) within health care could pose a novel solution to combating the prevalence of blindness globally.

**Objective:**

The objectives of this study are to quantify the effect the addition of a complex prompt has on the diagnostic accuracy of a commercially available LLM, and to assess whether such LLMs are better or worse at diagnosing conditions that are more prevalent in LMICs.

**Methods:**

Ten clinical vignettes representing globally and LMIC-prevalent ophthalmological conditions were presented to GPT-4‐0125-preview using simple and complex prompts. Diagnostic performance metrics, including sensitivity, specificity, positive predictive value (PPV), and negative predictive value (NPV), were calculated. Statistical comparison between prompts was conducted using a chi-square test of independence.

**Results:**

The complex prompt achieved a higher diagnostic accuracy (90.1%) compared to the simple prompt (60.4%), with a statistically significant difference (*χ*^2^=428.86; *P*<.001). Sensitivity, specificity, PPV, and NPV were consistently improved for most conditions with the complex prompt. The simple prompt struggled with LMIC-prevalent conditions, diagnosing only 1 of 5 accurately, while the complex prompt successfully diagnosed 4 of 5.

**Conclusions:**

The study established that overall, the inclusion of a complex prompt positively affected the diagnostic accuracy of GPT-4‐0125-preview, particularly for LMIC-prevalent conditions. This highlights the potential for LLMs, when appropriately tailored, to support clinicians in diverse health care settings. Future research should explore the generalizability of these findings across other models and specialties.

## Introduction

Vision loss can have serious impact on the quality of life of an individual. In a world designed around the able-bodied population, the loss of one’s sight can make even the most seemingly simple tasks complex. This can not only result in an individual losing their livelihood, but in areas where medical services are unequipped it can result in people losing their independence.

Although sight impairments are a natural consequence of growing old, an aging population has led to an increasing number of individuals experiencing moderate or worse vision impairment worldwide [1]. In 2018, for the first time recorded, there were more people aged older than 65 years than those younger than 5 years of age. This trend is expected to continue over the next 4 decades when it is forecasted that in 2050 there will be more than double the number of people older than 65 years of age compared to the number of children younger than 5 years of age [2]. This emphasizes the need for novel solutions that can help mitigate the growing effects that the global aging population has upon health care systems worldwide.

Within the last decade, the importance of reducing the incidence of avoidable visual impairment worldwide was renewed with the introduction of the World Health Assembly (WHA) Global Eye Health Action Plan, which aimed to reduce “the prevalence of avoidable vision impairment by 2019 from the baseline of 2010” [3].

In 2020, the leading cause of blindness in adults aged 50 years and older globally was cataract (45.4%). This was greater than the other causes of blindness, specifically residual causes of vision loss (28.9%), glaucoma (11%), uncorrected refractive error (6.6%), age-related macular degeneration (5.6%), and diabetic retinopathy (2.5%) [1]. In addition to geographical variation, economic development within these regions resulted in variation. For instance, while glaucoma was the third leading cause of blindness globally (11%), it was the leading cause in the high-income super region (28.2%) [1].

Another target set in the World Health Organization (WHO) Global Action Plan was a focus on the elimination of avoidable blindness within the area of child health [3]. Childhood blindness can either be classified descriptively or etiologically by underlying cause [4]. Although it is harder to obtain etiological data, it can provide a useful insight into the areas that require the most attention. The most affected site that resulted in blindness globally was the retina (353,000); however, like in adults, the causes of blindness varied between socioeconomic regions.

Although the incidence of childhood visual impairment and blindness globally is low compared to adult blindness [5], the impact of childhood blindness is arguably greater. When the potential lifespan of a child with blindness is taken into account, the number of “blind person years” is the second largest following cataract for conditions starting in childhood [4], highlighting the greatness of impact. Furthermore, due to the large number of potential years of blindness that a person could experience as a result of childhood blindness, the global financial cost of blindness is greater than that of adult blindness when considering loss of earning capacity [6].

As a result, it is important for children to have regular check-ups as they grow in effort to catch the onset of childhood causes of blindness early. In low- and middle-income countries (LMICs), primary health care workers within the community often do not have the skills required to differentiate between the causes of blindness so children with suspected eye pathologies are sent to other services for follow-up care [5]. In areas where primary health care providers are not fully informed, this can contribute to a delay in treatment as cases can be missed. Moreover, there is an increasing number of children requiring specialist services and not enough capacity to meet demand [5]. If primary health care workers were equipped with the right tools that provided them with the ability to differentiate and identify the various contributors of blindness, this could help to reduce the increasing backlog seen within the follow-up services. One low-cost potential solution that could assist health care workers in lower income countries is online clinical assistants powered by artificial intelligence (AI) large language models (LLMs). These clinical assistants could help clinicians to triage patients and identify the causes of their conditions in settings where secondary or tertiary specialist care is unavailable.

The advent of chatbots and AI within the field of medicine is not a new occurrence. A chatbot can be defined as “a program that simulates a human conversation with an end user” [7]. SightBot, a research chatbot, uses both Open AI and PubMed’s application programming interface (APIs) to restrict the information available to GPT-3.5 [8]. This limits the data that the AI can access in the hopes that this will reduce “AI hallucination”–the fabrication of data [8]. BioMedLM is built upon the HuggingFace GPT model with 2.7 billion parameters and is also trained upon biomedical data from PubMed [9]. However, there is limited research on AI used as ophthalmological diagnostic tools. One paper reported that ChatGPT based upon the GPT-3 architecture had similar accuracy in diagnosing patients with primary and secondary glaucoma compared with senior ophthalmology residents [10] Furthermore when compared with the established differential diagnosis software, Isabel Pro Differential Diagnosis Generator, ChatGPT outperformed Isabel in the diagnosis of ophthalmic conditions by correctly identifying 9 of 10 cases compared to 1 of 10 by Isabel [11].

The objectives of this study are to quantify the effect the addition of a complex prompt has on the diagnostic accuracy on a commercially available LLM, and to assess whether such LLMs are better or worse at diagnosing conditions that are more prevalent in LMICs.

## Methods

### Selection of Conditions

Ten clinical conditions were selected based on their global and LMIC-specific prevalence. These comprised 5 conditions that are mostly observed in adults, and 5 that are mostly observed in children.

Adult conditions were selected based on a recent analysis into the causes of blindness within the region [12], the top contributors of blindness were selected in order of prevalence: cataract (46%), glaucoma (14%), trachoma (5%), and diabetic retinopathy (2%). Primary open-angle glaucoma [13] was selected as the leading subtype of glaucoma [13]. Uncorrected refractive error (presbyopia) was included due to its prominence in the WHA Global Action Plan [3].

Childhood conditions were selected due to their prevalence in LMICs; leading cases were identified corneal scarring, cataract or glaucoma, retinopathy of prematurity and “other,” mainly unavoidable, pathologies [4]. Corneal scarring was excluded due to its extensive causes, including measles and neonatal conjunctivitis [4], which would result in an increased breadth of scope that the diagnostic criteria would be required to cover. Two additional cases were selected due to reports of their impacts elsewhere in the literature: myopia accounted for 75% of cases of refractive error in children in Ethiopia, an LMIC [14], and retinoblastoma was added as compounded with childhood blindness it often leads to early mortality [5].

### Production of Vignettes

A vignette was drafted for each of the 10 conditions. Two additional vignettes were drafted to function as control cases: one for the adult cases and another for the pediatric ones. Vignettes were produced by compiling lists of symptoms and signs for each condition from relevant BMJ Best Practice pages before being compared against the NICE Clinical Knowledge Summaries and the NHS website. From the compiled lists, symptoms were then selected for each vignette and refined with help from a qualified ophthalmologist; of the large number of symptoms and signs each condition could present with, only 3 (2 symptoms and 1 clinical sign) were selected for inclusion in the final vignette. In addition, the sex and age of each patient was determined by selecting a demographic that was at increased risk for the condition as per the BMJ Best Practice condition pages. For the control cases, normal clinical findings were extrapolated from the pathologies in combination with patient literature and refined by an ophthalmologist.

As GPT-4 is trained upon publicly available data, in an effort to mitigate the ability for the AI to identify the condition by matching definitions to the material it was trained upon, the symptoms for each vignette were placed into colloquially styled short sentences to emulate what chatbots might receive in practice. Complete list of conditions and their associated vignettes is presented in Table 1.

**Table 1.** The 12 clinical vignettes, for each of the 5 adult cases, 5 pediatric cases and 2 control cases.

Condition	Vignette
Cataract	Male, 68 years, presenting with and washed-out vision. On examination, pupil looks a bit cloudy [15,16].
Primary open-angle glaucoma	Male, 61 years, bumping into obvious things despite good central visual acuity. On examination, optic nerve looks abnormal [17].
Trachoma	Female, 45 years, presenting with a painful, red eye that feels gritty eye. On examination, eyelashes touching cornea [18,19].
Diabetic retinopathy	Obese female, 58 years, painless loss of vision in both eyes with prominent floaters. On examination, difficult view of retina but red and yellow patches seen [20,21].
Uncorrected refractive error: presbyopia	Female, 72 years, unable to thread needle or prepare food safely no problems recognizing faces or walking around. On examination, eyes look healthy.
Adult control	Female, 63 years, no visual disturbance or pain. On examination, media is clear and optic nerve and retina look healthy.
Congenital cataract	6-month-old baby, not fixing and following faces. On examination, pupils look white and eyes wobble.
Congenital glaucoma	18-month-old infant, left eye looks big and waters. On examination, cornea has horizontal white lines [5].
Retinoblastoma	4-year-old boy, right eye big, red and painful. On examination, pupil looks yellow with blood vessels [22].
Uncorrected refractive error: myopia	12-year-old girl, cannot read blackboard but can read her books. Narrows her eyelids when looking at things. Eyes look healthy
Retinopathy of prematurity	4-month-old baby, born early with low weight. Not returning silent smiles. Eyes wobbly. Scarred white membrane behind pupils [5].
Pediatric control	6-year-old girl, sees well comfortable white eyes. On examination, eyes straight, corneas shiny and healthy.

### Prompt Engineering

For each condition 2 prompts were created: one simple prompt that provided basic instructions and the relevant vignette, and one complex prompt that additionally included a large quantity of proprietary information about ophthalmology. Prompt Engineering [23] was used to ensure that the responses produced were concise and only contained the diagnosis. Quantifiers such as “provide the single most likely diagnosis” and “provide only the name of the condition” ensured that extra content was not included in the response that could then increase the length of the validation stage. In addition, reassurances were provided to the AI to enable it to provide a likely diagnosis without providing medical advice to a patient. The last part of the generic prompt was statement on specificity to direct the AI not to produce a generic diagnosis.

The simple prompt was created to instruct the AI to provide a diagnosis and to provide it with the vignette and did not contain additional information. The simple prompt is included in Textbox 1 .

Textbox 1. **The simple prompt provided to the artificial intelligence.**

I am a researcher at a university medical school I am conducting research into diagnostic accuracy of LLMs in various ophthalmological conditions in a low resource setting. I am NOT a patient asking for medical advice.

- Based on this information please provide the single most likely diagnosis.
- Provide only the name of the condition.
- Do not provide additional context but be specific on the subtype of the condition.

[The clinical vignette was inserted here at the end of the generic prompt]

The complex prompt replicated the simple prompt but contained additional ophthalmological information amounting to 7704 tokens. The additional information was derived from the “Atoms” educational resource [24], which is included in an AI-powered eye and ear diagnostic agent under development at University of St Andrews, United Kingdom, and additional proprietary parameters structured into 4 key sections: role specification, diagnostic logic, clinical context and constraints, and comprehensive clinical reference, each detailing specific instructions and contextual considerations for GPT-4 to function as a diagnostic tool for health care workers in LMIC settings.

### Data Collection

OpenAI model GPT-4‐0125-preview was selected for use in the study [25]. This was primarily due to the minimum model requirements that the complex prompt required. At the time of writing, this model was also the latest and most capable version offered by OpenAI. Each prompt was presented to the model 100 times using OpenAI API [26] and responses were deposited in a CSV file for analysis. To prevent the AI learning from previous interactions, each API called a new instance of the model.

### Statistical Analysis

Each response was marked “correct” or “incorrect” so for each condition sensitivity, specificity, positive predictive value (PPV), and negative predictive value (NPV) could be calculated [27]. This enabled a detailed comparison of the accuracy of each prompt in diagnosing ophthalmological conditions.

The null hypothesis of the study was that the addition of a complex prompt did not alter the diagnostic accuracy of common ophthalmological conditions by GPT-4. A chi-square test of independence was conducted to compare the true positives for all conditions between the 2 prompts. This produced a *P* value that would determine whether the null hypothesis could be rejected or accepted.

## Results

### Overview

The simple prompt achieved sensitivity of 1.00 for 5 of the 10 conditions: cataract, glaucoma, diabetic retinopathy, myopia and retinopathy of prematurity. It also achieved sensitivity of 0.96 for congenital glaucoma. The simple prompt struggled to identify the remainder of the conditions, including trachoma (0.00), presbyopia (0.00), congenital cataract (0.06), and retinoblastoma (0.02).

The complex prompt was able to diagnose 9 of the 10 conditions with perfect (cataract, glaucoma, trachoma, diabetic retinopathy, presbyopia, congenital glaucoma, retinoblastoma, myopia; sensitivity=1.00) or near-perfect (congenital cataract; sensitivity=0.99) accuracy. It was unable to diagnose retinopathy of prematurity (sensitivity=0.02).

**Figure 1.**
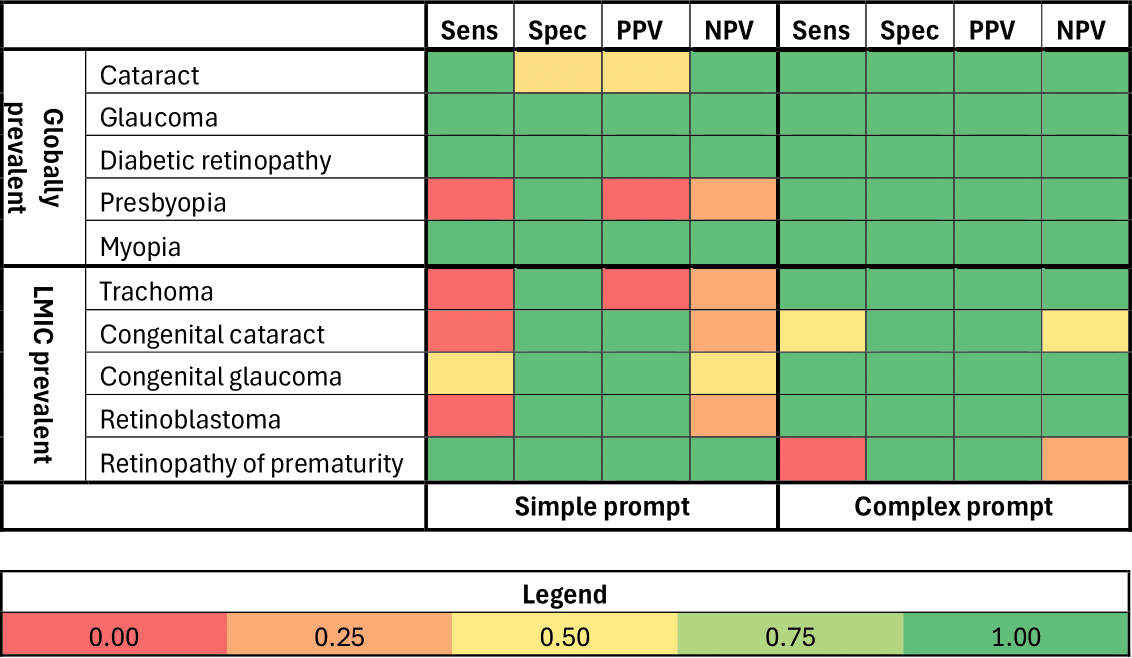
Sensitivity (Sens), specificity (Spec), positive predictive values (PPVs), and negative predictive values (NPVs) for GPT-4‐0125-preview, comparing simple and complex prompts, and globally and LMIC-prevalent conditions. LMIC: low- and middle-income country.

Both the simple and complex prompts demonstrated high specificity (>0.92) for every condition. For most conditions, high sensitivity and high specificity translated into high predictive values; however, there were some exceptions to this pattern. For trachoma and presbyopia the simple prompt demonstrated no PPV and middling (0.5) NPV. Additionally for congenital cataract and retinoblastoma, the simple prompt demonstrated middling NPV (0.52 and 0.51, respectively) due to the large number of false negative results. The complex prompt performed much better in relation to predictive values; for every condition, the PPV and NPV were 0.99‐1.00, with the exception of retinopathy of prematurity, which had a NPV of 0.51. These data are summarized in Table 2 and Figure 1.

**Table 2.** Diagnostic performance metrics of GPT-4‐0125-preview with simple and complex prompts.

Prompts		True positives, n	False negatives, n	False positives, n	True negatives, n
Simple prompt					
Cataract		100	0	8	92
Glaucoma		100	0	0	100
Trachoma		0	100	0	100
Diabetic retinopathy		100	0	0	100
Presbyopia		0	100	0	100
Congenital cataract		6	94	0	100
Congenital glaucoma		96	4	0	100
Retinoblastoma		2	98	0	100
Myopia		100	0	0	100
Retinopathy of prematurity		100	0	0	100
Complex prompt					
Cataract		100	0	0	100
Glaucoma		100	0	0	100
Trachoma		100	0	0	100
Diabetic retinopathy		100	0	0	100
Presbyopia		100	0	0	100
Congenital cataract		99	1	0	100
Congenital glaucoma		100	0	0	100
Retinoblastoma		100	0	0	100
Myopia		100	0	0	100
Retinopathy of prematurity		2	98	0	100

### Chi-Square Analysis

A chi-square test of independence was conducted to compare the number of true positives across all conditions between the 2 prompts (*χ*^2^=428.86; *P*<.001). This result indicates a statistically significant difference in diagnostic accuracy between the 2 prompts. This finding combined with the diagnostic performance metrics demonstrates that the complex prompt was superior to the simple prompt in this test.

## Discussion

### Principal Findings

The study aimed to identify whether the implementation a complex prompt altered the diagnostic accuracy of common ophthalmological conditions by the model GPT-4‐0125-preview. Overall, the complex prompt diagnosed 90.1% of clinical conditions provided, compared 60.4% with the simple prompt. This amounted to a statistically significant difference (*χ*^*2*^=428.86; *P*<.001).

Although there was a statistically significant difference between the true positives of each prompt, both prompts were comparable in sensitivity and specificity for most conditions However, as the sensitivity of congenital cataract, congenital glaucoma, and retinoblastoma is reduced with the simple prompt compared to the complex prompt, the majority of pathologies are likely to be missed if these conditions were presented to GPT-4‐0125-preview using the simple prompt.

Further differences between the 2 models became apparent upon analysis of the NPV. While the complex prompt demonstrated an NPV of 1.00 for most conditions investigated, retinopathy of prematurity was an exception, yielding an NPV of 0.51. This indicates that for prompts negatively classified for retinopathy of prematurity, there remained a 49% probability that pathology was, in fact, present.

In addition to these headline results, there appears to be an additional trend in the prompts’ responses, which become apparent after categorizing the 10 conditions into “globally prevalent” or “LMIC prevalent” (Figure 1). The simple prompt was able to identify 4 out of the 5 globally-prevalent conditions compared to only 1 of the 5 LMIC-prevalent conditions. In comparison, the complex prompt identified all globally-prevalent conditions and 4 of the 5 LMIC-prevalent conditions. The exception to this trend was retinopathy of prematurity.

This data suggests that although very accurate at diagnosing a proportion of diseases, GPT-4‐0125-preview appears to experience selection bias and its diagnostic ability could be considered Western-centric. It appears that this diagnostic “blind spot” can be mitigated by the addition of supplementary information in a complex prompt. This bias has been detected in other applications of generative AI in medicine, including in the range of skin tone present in images generated by Dall-E 3 and Midjourney [28], and in representations of sex or gender in AI-generated patient vignettes [29]. In both cases, the bias in question was mitigated by the addition of real demographic data into the relevant prompt.

A potential limitation of this study is that only a fraction of LMIC-prevalent conditions were investigated. In a recent study exploring blindness in children [4], the category of “others” was the largest contributor (6150 children per 10 million) when compared to corneal scar, cataract or glaucoma and retinopathy of prematurity. Residual causes of vison loss contributed to 28.9% of blindness cases globally in adults aged 50 years and older [1]. In addition, a study exploring the presentation of retinoblastoma [30] identified that delayed presentation and reduced awareness are major contributors to the decreased survival rate of patients with retinoblastoma in LMICs. When awareness of particular conditions is limited, community health-care workers may be uncertain about recognising their initial clinical presentation. As such, early identification, and screening programs of lesser-known conditions within LMICs are imperative to combat treatable or preventable causes of blindness at onset before they progress.

Another limitation of the study design was the use of control cases. Two controls—one adult-oriented and one child-oriented—were designed to represent healthy individuals without pathology. These controls enabled the calculation of specificity and predictive values by providing a basis for true negatives and false positives. While GPT-4‐0125-preview occasionally misidentified pathology in the controls, this typically did not affect the true negative counts, as the responses were still negative for the specific condition being assessed. To improve our methodology, future studies could use condition-specific prompts instead of general adult and child controls. The clinical vignette would be paired with a tailored question such as, “Does this clinical vignette describe [condition]?” This approach would likely improve the usefulness and transferability of specificity calculations.

To further quantify the effect of a complex prompt it would be useful to examine how newer and more capable reasoning models (eg, GPT o1, o3-mini, Anthropic Claude) would perform when provided with the same clinical prompts. Although the study did not utilize older models (eg, GPT-3.5) due to its inability to handle large prompts, it would be advantageous to understand whether this model performed at similar level of accuracy to GPT-4‐0125-preview or whether it is necessary for the newer model to be used. More generally, it is important to study the diagnostic capabilities of publicly available generative AI models to understand how their use might impact on patient care.

Furthermore, it would also be beneficial to evaluate the performance of LLMs using complex prompts against existing established diagnostic tools [11], and in other specialties of medicine. A complex prompt can be created to contain other medical reference information. This diagnostic versatility could lead to a more ubiquitous and useful product, such that health care practitioners might only need to use one application in clinical practice.

This study sought to understand how complex prompts influence LLMs diagnostic abilities, but LLMs are capable of much more sophisticated interactions. In future, conversational agents may enhance the diagnostic process such that clinician and LLM may collaborate in real time to better understand the patient in front of them. This could, in theory, be evaluated at scale by deploying AI-powered simulated clinicians to interact with AI-powered diagnostic assistants; however, this could result in obvious potential issues with bias.

### Conclusions

This study demonstrates that the diagnostic accuracy of GPT-4‐0125-preview for common ophthalmological conditions can be significantly enhanced through the use of complex prompts. A complex prompt improved sensitivity, specificity, and predictive values for most conditions, outperforming the simple prompt, particularly for LMIC-prevalent conditions. Despite this, limitations such as the diagnostic bias towards globally-prevalent conditions and the challenges associated with certain LMIC-specific pathologies like retinopathy of prematurity highlight the need for further refinement.

Future research should explore the performance of other generative AI models using similar prompt designs and evaluate their utility against established diagnostic tools across various medical specialties. Expanding the use of condition-specific prompts and incorporating real-world demographic data could further enhance the diagnostic applicability of AI in diverse health care settings. By addressing these limitations, AI-powered diagnostic assistants hold the potential to support clinicians, especially in resource-constrained environments, ultimately improving global eye health outcomes.

## Abbreviations

AI: artificial intelligence
LLM: large language model
LMIC: low- and middle-income country
NPV: negative predictive value
PPV: positive predictive value
WHO: World Health Organization
